# Postmortem diagnostics of assumed suicidal food anaphylaxis in prison: a unique case of anaphylactic death due to peach ingestion

**DOI:** 10.1007/s12024-021-00373-1

**Published:** 2021-05-03

**Authors:** Stefano Tambuzzi, Guendalina Gentile, Michele Boracchi, Domenico Di Candia, Rachele Bianchi, Riccardo Zoja

**Affiliations:** Dipartimento di Scienze Biomediche per la Salute, Sezione di Medicina Legale e delle Assicurazioni, Università degli Studi di Milano, Via Luigi Mangiagalli, 37 - 20133 Milano, Italy

**Keywords:** Food anaphylaxis, Peaches, Suicide, Prison, Tryptase, Salicylates

## Abstract

Suicidal ingestion of food which the victim is aware they are allergic to is an exceptional occurrence in the forensic field. To the best of our knowledge, no cases of suicidal food anaphylaxis have been reported to date. Therefore we present the first case described in the literature. A 30-year-old prisoner was found dead inside his cell with the remains of a peach remains next to his body, and a handwritten farewell note in his pocket. The autopsy revealed only non-specific findings, while laboratory investigations (serological, toxicological, histological, and immunohistochemical) played a pivotal role in determing the cause and manner of death. In particular, a high titer of both total and specific IgE antibodies was detected, as well as an increase of the tryptase level in cadaveric blood. Moreover, a massive concentration of salicylates was measured in the gastric contents. Microscopically, cellular residues characterized by a vegetal structure were observed in the gastric contents and elements suggestive of mast cells were detected in the glottis, lungs, and myocardium. The immunohistochemical investigation with anti-CD117 and anti-tryptase antibodies showed positivity for mast cells, some of which appeared degranulated. Such findings were entirely consistent with an acute systemic anaphylactic reaction triggered by allergy. Therefore, the prisoner’s death was attributed to self-induced food anaphylaxis caused by the ingestion of peaches. This conclusion was achieved based only on circumstantial data, anamnestic information, autopsy findings, and multiple laboratory results. This integrated approach should be used to pursue a post-mortem diagnosis of anaphylaxis.

## Introduction

Among all fatalities, deaths due to allergic phenomena represent a small sub-category. Since anaphylactic shock can cause death within few minutes if not properly treated [1], cases of fatal anaphylaxis are frequently subjected to investigations by forensic pathologists, especially when deaths are unwitnessed. Furthermore, sudden anaphylactic fatalities that occur inside a hospital, sometimes in the emergency room, or under medical treatment, are also usually subjected to forensic autopsies [2]. Anaphylactic shock is usually associated with accidental events [3] rather than suicide [4]; as far as we know, it has never been indicated in a homicide. It is well-recognized that macroscopic autopsy findings, such as organ congestion and soft tissue edema [5], as well as conventional histology examination, are not necessarily specific for the diagnosis of lethal anaphylaxis [6, 7]. For this reason, the forensic diagnosis of lethal anaphylaxis is often based on the exclusion of other possible causes, such as asphyxiation, acute drug toxicity, traumatic death, and other life-threatening diseases, in combination with a history of allergy [8, 9]. Therefore, the diagnosis is generally based to some degree on circumstantial evidence that supports the existence of a causal link between the victim’s death and the ingestion of certain medications or foods (dried fruit [10], shellfish [11], fresh fruit [12]), as well as animal bites or stings [2]. The victim's medical history can thus lead to a suspicion of anaphylaxis, but it is essential to obtain further evidence of anaphylaxis, especially in deaths of forensic concern [13].

We present the first case to be described in the literature of suicide that occurred in prison through assumed voluntary food anaphylaxis. The absence of autopsy findings clearly diagnostic for anaphylaxis led us to investigate this hypothesis further through serological, toxicological, histological, and immunohistochemical analyses. We, therefore, report the results and discuss this case’s peculiar features.

## Case Report

A 30-year-old Albanian man, who had been detained for seven years in prison in Milan on charges of drug dealing and possession, was found dead inside his cell. It was not a high-security cell and it could accommodate two people, but the prisoner had been alone for two days as a new detainee had not yet arrived (the decedents previous cell mate had been released). Food remains were found next to the body and they were identified as partially eaten peaches with exocarp. To clarify what happened, the Investigating Magistrate decided to start forensic investigations. It was discovered that the victim, having maintained good behavior whilst in prison, enjoyed some benefits such as the possibility of being involved in work activities. In particular, he was in charge of cleaning the canteen and the other common areas inside the penitentiary structure. Analysis of the victim's clinical documentation revealed that the man was a former drug addict and heavy smoker, with a history of asthma attacks and allergy to some pollen not otherwise specified. There was not any mention of food allergies. The last psychological evaluation was carried out one year earlier and it had revealed a moderate mood deflection, without any previous suicidal ideation or attempt. The man was not under pharmacological treatment. By the order of the Investigating Magistrate, a judicial autopsy was performed three days later.

## Forensic investigations

### Gross examination

The man's body was quite well-nourished and in a good state of preservation (weight: 57 kg; length: 176 cm). He was wearing a prison jumpsuit. A handwritten note, which read “… *I’m done with this!… I’m allergic… I ate something I know it will kill me….*” was found in his pocket. The external examination did not reveal any signs of traumatic injuries. Only some black tattoos and several scars related to previous and repeated drug injections were detected on both arms. There were no recent signs of venipuncture. By inspecting the oral cavity, mild hyperemia of the oral and labial mucosa was observed. The autopsy examination revealed pulmonary, hepatic, and bilateral renal congestion, as well as liquid blood and reddish mucus in the tracheal and bronchial lumen. Edema of the glottis, urticaria, or other skin reactions typical in allergic reactions were not found. Upon the opening of the stomach, 150 ml of gastric contents with a brownish and corpuscular appearance were documented with partially digested food fragments consisting of laminar specimens similar to cuticles with a vegetal appearance and leathery consistency. Such identifiable components of gastric contents appeared similar to the peaches exocarp that had been found next to the body. No food material was found in the airways and no pathological findings were observed on all the other viscera. Samples from different organs (brain, cerebellum, brainstem, tongue-larynx-thyroid block, first tracheal rings, esophagus, lungs, bronchi, whole heart, liver, spleen, kidneys, and pancreas) as well as gastric contents were collected for histological investigations. Similarly, biological fluids and organs (cardiac and femoral blood, bile and urine, brain, lungs, liver, and kidneys) were collected, as well as hair, gastric contents, and nasal swabs for subsequent chemical-toxicological investigations. At the conclusion of the gross examination, the cause of death remained undetermined, since evidence of potentially lethal illnesses could not be found. In particular, the suspicion of a lethal anaphylactic shock, which was based on circumstantial data (food remains found next to the body and the handwritten note), was not either confirmed or excluded. Therefore, the Investigating Magistrate authorized the execution of post-mortem laboratory analyses and we decided to perform serological, toxicological, histological, and immunohistochemical examinations aimed at searching for the main markers of anaphylaxis. Finally, he also authorized the comparison between the writing found on the handwritten note and the known handwriting of the victim; a perfect match was observed.

### Serological examination

Post-mortem IgE tests brought forward a high titer of IgE antibodies (154 kU/l) in the patient’s femoral blood. The standard range of IgE was indicated as < 100 kU/l for adults by the analyzing laboratory. Further tests revealed moderately increased to very high concentrations of specific IgE against peach and birch tree antigens (Table 1).

Additional tests for serum tryptase concentrations demonstrated a high serum level of the enzyme of 15.7 μg/l. The normal range of tryptase in serum, i.e. the reference value in healthy individuals, is indicated in the literature between 5.6 and 9.8 μg /l (a value of the 90th percentile) [14]. Tryptase concentration in the patient in question had never been measured before; therefore an acute rise of the enzyme blood concentration due to mast cell degranulation and pathognomonic in anaphylaxis, could not be proved beyond doubt.

### Toxicological examination

All the analyses were carried out at the Laboratory of Forensic Toxicology of the University of Milan. The samples were tested to identify the presence of illicit drugs, alcohol, or other substance with pharmacological activity. Toxicological investigations were negative for both the blood alcohol content and substances of abuse. The man had only traces of caffeine and cotinine, which were substances of no toxicological significance. The literature suggests that if food anaphylaxis is suspected, gastric contents should be collected to confirm that a certain food allergen was ingested before death. However, the search and identification of specific food allergens in cadaveric gastric contents have always had a very poor success [15]. For this reason, we preferred to conduct another analysis, aimed at analysing for salicylic acid in the gastric contents, as it may be significant for the ingestion of peaches. Indeed, peaches, along with plums, are particularly rich in salicylic acid, with medium values of 43.95 µg per 100 g of food [16], especially in the exocarp. A sample of gastric contents was therefore analyzed using HPLC / MS–MS Q Exactive Orbitrap Hybrid Mass Spectrometer (Thermo Fisher Scientific). The findings were acquired using Thermo Xcalibur™ software and the salicylates were dosed. A massive presence of this substance (130 ng/L) in the victim’s gastric contents was detected.

### Histological examination

**Fig. 1:**
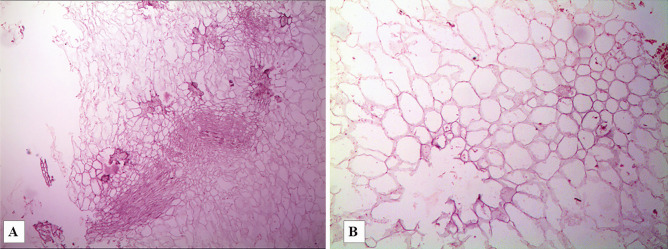
Gastric contents – On the left **a**: a microscopic view of cellulose fragments with quadrangular/rectangular cavity structure, without evidence of cell residues (H&E, 50x). On the right **b**: a higher magnification (H&E 100x) shows the regular structure of the cellulose scaffolding residues

After standard histological processing with post-fixative techniques and Hematoxylin–Eosin staining, all samples were observed using optical microscopy. The glottis, especially the right side, was affected by moderate chronic inflammatory cellular infiltrates, consisting of lymphocytes, plasma cells, and elements suggestive of mast cells. Vascular dilatation and congestion, with modest edematous imbibition of the glottal subepithelial connective tissue, were also observed. The lungs and both the tracheal and bronchial mucosa showed cellular elements suggestive of mast cells; the same finding was documented in the anterior and posterior walls of the left ventricle, as well as the interventricular septum. The microscopic analysis of the gastric contents revealed cellular residues characterized by a vegetal structure, with cavitated cellulose scaffolding (Fig. 1). Due to their morphology and appearance, they were identified as peach residues. This finding was consistent with both the circumstantial data and the presence of salicylic acid in the gastric contents.

### Immunohistochemical examination

**Fig. 2:**
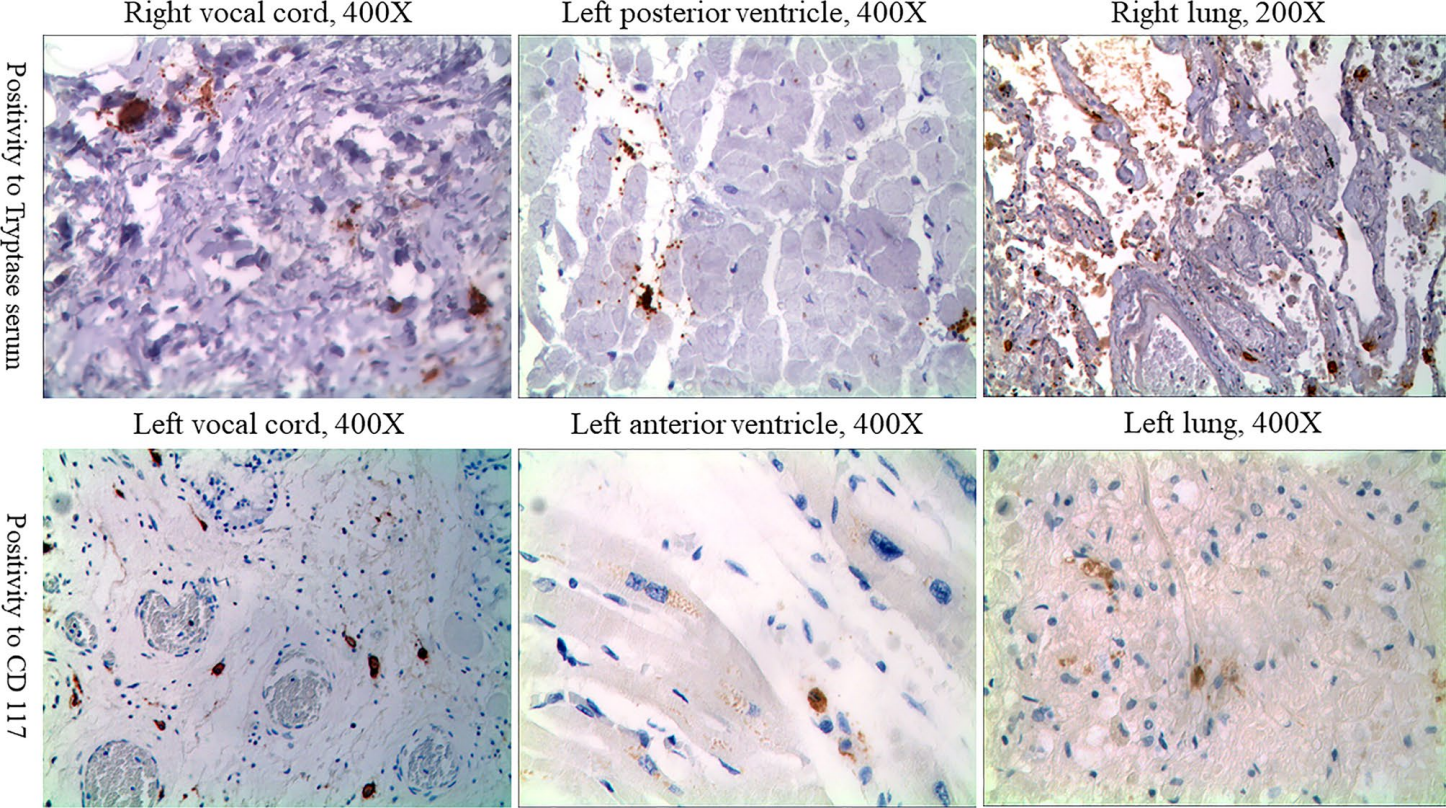
Microscopic views of the tissue’s positivity to immunohistochemical staining with both anti-tryptase (upper row) and anti- CD 117 (lower row) antibodies; it was performed on the glottis (right and left vocal cords), myocardium, and lungs samples

In light of the histological findings, we deemed it essential to try to make visible acute mast cell degranulation in the victim's tissues. For this purpose we selected samples of glottis, lungs, and myocardium; all of them had already shown cellular elements suggestive of mast cells with H&E staining. On such samples, immunohistochemical staining was performed using anti-CD 117 antibodies and especially anti-tryptase antibodies. A fair number of cells with cytoplasm rich in granules reactive to both the applied antibodies was documented in the glottis; such finding was consistent with the presence of not degranulated mast cells in the subepithelial connective tissue between the glands, and the connective tissue between the striated muscular fibers of the true vocal cord. Also, several cells with a rounded nucleus and a large cytoplasm but without granules were observed; they were considered entirely consistent with degranulated mast cells. Around them, numerous anti-tryptase positive granules were scattered in the subepithelial connective tissue and between the muscle fibers, with a starry sky-like or yard-like pattern of distribution. Similar findings were observed in lung and myocardium samples. In both of them, a positive reaction for both anti-tryptase and anti- CD 117 was documented. Such findings were more conspicuous in the lung samples than in the myocardium samples (Fig. 2). Overall, these immunohistochemical findings were consistent with an acute degranulation of mast cells indicating an acute systematic reaction triggered by allergy [13].

At the end of all the above mentioned forensic investigations and based on their mutual consistency, the victim’s cause of death was assumed to be anaphylaxis.

## Discussion

The severe and potentially lethal multi-organ hypersensitivity reaction [17] defined as anaphylactic shock is due to the exposure to a specific allergen [9, 18], towards which an immune system sensitization has already developed. The re-exposure to the same allergen—type I hypersensitivity IgE antibodies mediated—ultimately causes the degranulation of the mast cells and of the tissue basophils with the release of histamine and other mediators that may cause a precipitous death due to cardio-respiratory arrest [7]. However, the complexity of the pathogenic factors and pathophysiological processes involved in anaphylaxis, make a post-mortem diagnosis extremely complex [7]. When approaching suspected cases of lethal anaphylaxis, forensic pathologists must rely on the evaluation of circumstances surrounding death [15], as well as the information acquired from the victim's clinical history [7]. However, if these elements are lacking or completely absent, expressing a judgment of certainty would be almost impossible. In this regard, the lack of specific and reliable markers [19] for the post-mortem diagnosis of anaphylaxis also contributed to diagnostic difficulty and very often diagnosis of lethal anaphylaxis requires the use of the exclusion criteria [7]. Since episodes of lethal anaphylaxis may include forensic cases in which medical management has been absent, insufficient or delayed [19], it becomes more essential than ever to obtain further evidence of anaphylactic shock [13].

Although the autopsy findings associated with anaphylaxis may sometimes be suggestive of it, they are neither specific nor essential [15] and they can also vary in relation to the type of involved allergen, the type of administration, as well as the time that elapses between the onset of the allergic reaction and death. In particular, it has been reported that in immediate anaphylactic deaths (sudden cardiovascular collapse) [20], significant autopsy findings may not be present and only a non-specific plurivisceral congestion may be detected [2].

The post-mortem diagnosis of anaphylaxis differs profoundly from that in a clinical setting, which is typically supported by an increase in serum proteases within two hours of the acute event [21]. Tryptase is a protease secreted by degranulating mast cells in the blood and interstitial space as a consequence of an acute allergic reaction [14]. However, the victim’s individual baseline tryptase level cannot be evaluated *ex post*. Moreover, such protease level in cadaveric blood may not be relied upon the same degree of evidence and reliability as in clinical examination for multiple factors [14]: i) influence of post-mortem autolysis and PMI with antigen masking and elevation of serum proteases [2, 15], ii) other morbid conditions, such as myocardial infarction [22], parasitosis with hypereosinophilia [23] and mastocytosis [9, 20, 24], iii) multiple trauma [25], asphyxia [26], heroin poisoning [20, 27], iv) blood sampling site [20] and v) lack of standardized cut-offs [20]. Given such difficulties of interpretation, only very high post-mortem values of tryptase are accepted in the literature as significantly pivotal for a death resulting from an anaphylactic reaction [10]. In contrast, the total amount of IgE antibodies in cadaveric blood—even at average room temperature—is relatively stable after death and it can be considered as a reliable parameter associated with allergy [14]. In particular, the combination of specific IgE antibodies with tryptase levels can facilitate the differential diagnosis and significantly improve the diagnostic accuracy in post-mortem scenarios [9].

As far as the diagnostic value of the mast cells related to hypersensitivity, a univocal position is lacking in the literature, since they are not only normally present in all healthy tissues [26], but also difficult to count in anaphylaxis [7] and with degranulation challenging to identify on cadaveric material [2]. Recent studies have focussed on the importance and specificity of the accumulation of mast cells and eosinophils in specific organs such as the spleen [28], intestine, lungs, and throat mucosa [8, 14].

In the case presented here, circumstantial data—food remains found next to the body and the handwritten note—led us to hypothesize that the death was potentially due to food anaphylaxis. The victim's clinical documentation only reported a previous history of drug addiction, asthma attacks, and allergy to some pollen not otherwise specified; information about food allergies was not reported. The forensic autopsy first of all ruled out the presence of signs attributable to blunt force trauma or stab wounds, as well as the existence of a food bolus in the airways. Only mild hyperemia of the oral and labial mucosa and pulmonary, hepatic, and bilateral renal congestion, as well as liquid blood and reddish mucus in the tracheal and bronchial lumen, were observed. Such findings are undoubtedly non-specific but still consistent with death due to anaphylaxis, even if neither edema of the glottis nor skin reactions typical in allergy were detected. The subsequent laboratory tests were pivotal to investigate the existence of markers of anaphylaxis. The serological analysis revealed a high titer of both total and specific IgE antibodies, which is a sign of the victim's high allergic predisposition. Given the discovery of peach remains next to the body, we firstly decided to assess the specific IgE antibodies for the main peach allergens (rPru p 1, rPru p 3, and rPru p 4) and secondly the specific IgE antibodies for the main birch tree allergens ( rBet V 1, rBet V2). The latter were investigated for two reasons: the victim was allergic to some pollen not otherwise specified and it has been reported in the literature that a cross hypersensitivity reaction between peach and birch tree allergens exists [29–31]. Overall, specific IgE antibodies were found to be moderately or markedly elevated. Serum tryptase level was also significantly increased, indicating a specific mast cell activation, although it may also partly be due to the 3-day post mortem interval (PMI).

To assess any eventuality, toxicological analyses were performed. They only highlighted a massive concentration of salicylates in the gastric contents. In this regard, we ruled out that the victim had taken salicylate-based drugs over the hours before death as no mention was reported in the medical record and the thorough search of the cell did not reveal any concealed substance. Therefore, the awareness that peaches are particularly rich in salicylates [16] led us to believe that the presence of these substances in the gastric contents may be attributed to the intake of such fruits. This assumption was corroborated by the partially eaten peaches with exocarp found next to the body. Finally, we resorted to histological and immunohistochemical analyses. The first ones showed cellular residues characterized by a vegetal structure in the victim’s gastric contents; besides, the presence of elements suggestive of mast cells was highlighted in the glottis, lungs, and myocardium. On the same samples, immunohistochemical investigation with anti-CD117 and anti-tryptase antibodies revealed a fair number of mast cells, some of which contained a high number of positive granules. At the same time, the vacuolar accumulation of anti-tryptase positive material around the mast cells was interpreted as a sign of degranulation. These findings were entirely consistent with an acute systemic anaphylactic reaction triggered by allergy. Obviously, we point out that the post-mortem tissue autolysis may act as a potential interference factor, by altering the total amount of detectable mast cells [2]. However, in our case the combination of circumstantial data, anamnestic information, autopsy, and laboratory findings (serological, toxicological, histological, and immunohistochemical), as well as their mutual consistency, allowed us to reasonably attribute the prisoner’s death to a food anaphylactic shock [20]. In detail, high titers of both serum tryptase and IgE antibodies were detected. As it is known, since such antibodies are relatively stable after death [9, 14], they provided significant diagnostic assistance, especially after being integrated with the history and other post-mortem findings.

To define the manner of death we mainly relied on circumstantial data and autopsy findings, which were corroborated by laboratory results. Indeed, no evidence of homicide was found. It could have been an accidental death, however, the circumstantial data made this hypothesis extremely unlikely. It was in fact a young man in prison for seven years, with a moderate mood deflection which was not being pharmacologically treated. Moreover in a pocket of the decedents prison jumpsuit a handwritten note with explicit content was found. The note had been written by the victim himself, as demonstrated by a comparison with the known handwriting of the prisoner. Based on such pivotal evidence, we concluded that the man committed an unusual suicide by ingesting food which he was aware he was allergic to. The clinical information in our possession was provided by the victim himself and we are not aware of whether or not the man had experienced severe allergic episodes after ingestion of peaches, before being detained in prison. It is possible that this piece of information was not deliberately reported by the victim to the clinicians. However, we can affirm that the victim was intimately convinced and aware of the high probability of dying following the ingestion of peaches, despite the indirect and not necessarily lethal action of the applied mean. The farewell note is in fact explicit in such a sense. The man not only claimed to be tired of his own living conditions but also to have ingested something that would have killed him (“… *it will kill me*”). Self-induced food anaphylaxis represents a rare manner of suicide, hitherto not reported in the literature. Forensic pathologists should therefore always keep in mind that subjects aware of suffering from a food allergy can voluntarily ingest food for suicidal purposes. Such cases, as every death following acts of commission and omission acting via indirect means, are tricky scenarios in which the establishment of the cause and manner of death is far from straightforward.

In our case, since the man’s death occurred in prison, it was more essential than ever to unequivocally demonstrate the real cause of death. In the face of a suspicion based on circumstantial data and of not conclusive autopsy findings, the serological and immunohistochemical analyses proved to be pivotal. At the same time, histological and toxicological investigations also unexpectedly provided a significant contribution, by highlighting respectively vegetal residues and a massive concentration of salicylates in the gastric contents. However such information was useful only after being integrated with the circumstantial data; otherwise, in fact, they would most likely have been of little forensic relevance. Overall, the post-mortem diagnosis of anaphylaxis can thus properly be pursued only after the integration of multiple coherent and convergent forensic findings, each of them may be considered as a piece of a single puzzle.

## Key points

1. Suicidal food anaphylaxis is a rare occurrence, especially in prison.
2. For forensic pathologists the post-mortem diagnosis of lethal anaphylaxis is potentially complex and challenging.
3. Circumstantial data is typically of value in the assessment of possible lethal anaphylaxis.
4. Laboratory analyses are essential to pursue the post-mortem diagnosis of anaphylaxis.
5. An integrated approach is pivotal when dealing with forensic cases of anaphylaxis.

**Table 1:** Determination of specific IgE concentrations in cadaveric femoral blood samples

Allergen	Specific IgE concentration (kUA/l)	Reference value (kUA/l)
rPru p 1 (peach -*Prunus Persica*)	0.09	0.10
rPru p 3 (peach -*Prunus Persica*)	0.76	0.10
rPru p 4 (peach -*Prunus Persica*)	0.19	0.10
rBet V 1 (birch tree – *Betula Verrucosa*)	0.53	0.10
rBet V 2 (birch tree – *Betula Verrucosa*)	0.44	0.10
